# No impact of weather conditions on the outcome of intensive care unit patients

**DOI:** 10.1007/s10354-021-00830-0

**Published:** 2021-03-18

**Authors:** Raphael Romano Bruno, Bernhard Wernly, Maryna Masyuk, Johanna M. Muessig, Rene Schiffner, Laura Bäz, Christian Schulze, Marcus Franz, Malte Kelm, Christian Jung

**Affiliations:** 1 Division of Cardiology, Pulmonary Diseases, and Vascular Medicine, Medical Faculty, University Hospital Düsseldorf, Moorenstraße 5, 40225 Düsseldorf, Germany; 2 Clinic of Internal Medicine II, Department of Cardiology, Paracelsus Medical University of Salzburg, Salzburg, Austria; 3 Division of Cardiology, Department of Medicine, Karolinska Institutet, Karolinska University Hospital, Stockholm, Sweden; 4 Department of Neurology, Jena University Hospital, Friedrich Schiller University, Jena, Germany; 5 Orthopedic Department, Jena University Hospital, Friedrich Schiller University, Jena, Germany; 6 Department of Internal Medicine I, Division of Cardiology, Angiology, Pneumology, and Intensive Medical Care, University Hospital Jena, Friedrich-Schiller-University, Jena, Germany; 7 Cardiovascular Research Institute Düsseldorf (CARID), Düsseldorf, Germany

**Keywords:** Sudden weather changes, Intensive care unit, Outcome, Mortality, Weather conditions, Plötzliche Wetteränderungen, Intensivpflege, Outcome, Mortalität, Wetterbedingungen

## Abstract

Global warming leads to increased exposure of humankind to meteorological variation, including short-term weather changes. Weather conditions involve changes in temperature, heat and cold, in air pressure and in air humidity. Every single condition influences the incidence and mortality of different diseases such as myocardial infarction and stroke. This study investigated the impact of weather conditions on short- and long-term mortality of 4321 critically ill patients (66 ± 14 years, 2638 men) admitted to an intensive care unit (ICU) over a period of 5 years. Meteorological information (air temperature, air pressure and humidity) for the same period was retrieved. The influence of absolute weather parameters, different seasons, sudden weather changes including “warm” and “cold” spells on ICU and long-term mortality was analyzed. After correction for Simplified Acute Physiology Score (SAPS-2), no impact of meteorological conditions on mortality was found. Different seasons, sudden weather changes, “warm spells” or “cold spells” did not affect the outcome of critically ill patients.

**Supplementary Information:**

The online version of this article (10.1007/s10354-021-00830-0) contains supplementary material, which is available to authorized users.

## Abstract (de)

Die globale Erderwärmung führt dazu, dass die Menschheit vermehrt meteorologischen Schwankungen, einschließlich kurzfristiger Wetteränderungen, ausgesetzt ist. Zu diesen Wetterbedingungen gehören Veränderungen der Temperatur, der Hitze und Kälte, des Luftdrucks und der Luftfeuchtigkeit. Jeder dieser Faktoren beeinflusst die Inzidenz und Mortalität verschiedener akuter Erkrankungen wie Herzinfarkt und Schlaganfall. Diese Studie untersucht den Einfluss der Wetterbedingungen auf die Kurz- und Langzeitmortalität von 4321 kritisch kranken Patienten (66 ± 14 Jahre, 2638 männlich), die über fünf Jahre auf eine Intensivstation (ICU) eingewiesen wurden. Es wurden meteorologische Informationen (Lufttemperatur, Luftdruck und Luftfeuchtigkeit) für den gleichen Zeitraum erfasst. Der Einfluss absoluter Wetterparameter, verschiedener Jahreszeiten, plötzlicher Wetteränderungen einschließlich „warmer“ und „kalter“ Perioden auf die ICU- und Langzeitmortalität wurde analysiert. Nach Korrektur für SAPS‑2 wurde kein Einfluss der meteorologischen Bedingungen auf die Mortalität gefunden. Unterschiedliche Jahreszeiten, plötzliche Wetterumschwünge, „Hitzeperioden“ oder „Kälteperioden“ hatten keinen Einfluss auf die Prognose kritisch kranker Patienten.

## Introduction

Several external parameters can influence the outcome of patients who are admitted to an intensive care unit (ICU). Beginning in early antiquity, Hippocrates (430 BC) observed in his treatise “Of Airs, Waters, and Places” that environmental factors influence the pathogenesis of the disease. Today, there is an ongoing discussion that human health may be very sensitive to sudden weather changes. Now, humankind faces climate change that poses a considerable threat to the environment, the global economy, social cohesion, and—of course—healthcare. Global warming leads to increased exposure of humankind to extreme meteorological variation. Measurable consequences for health can already be detectable as growing data indicates that weather conditions affect admission rates and mortality [1]. In fact, sudden cold or warm waves are thought to increase myocardial oxygen consumption causing cardiac arrhythmias or angina attacks [2]. In addition, cold temperatures lead to an increased release of catecholamines, which results in sympathomimetic stress [3]. Weather effects can be divided into changes in temperature, heat and cold, into air pressure changes and into changes in humidity.

Extensive epidemiologic studies demonstrated a relationship between mortality and cold temperatures during winter or heatwaves [4, 5]. Out-of-hospital cardiac arrest occurs more often during winter [6]. This applies especially to cardiovascular diseases (CVD): Rapid weather changes lead to a significantly increased emergency department visits due to CVD [7, 8]. Acute myocardial infarction occurs more often during winter and spring and less during summer in Japan [9], USA, [10], Sicily [11], and Greece [12]. Younger males are more affected by coronary heart disease during spring and mature males during winter. Furthermore, sudden pressure drops in winter are associated with a significant excess in cardiovascular disease mortality and a substantial rise in hospital admissions [13, 14]. This effect is also true for ischemic heart disease with an age-dependent peak. Younger patients are more sensitive to cold spells, while patients over 65 years seemed to be more affected by hot waves [15]. For heatwaves, several countries and regions developed Heat–Health Warning Systems that can reduce mortality during heat seasons. Of note, seasonal patterns influence even suicide mortality rates [16].

Most of the epidemiologic data is derived from registers. The present study investigates whether these observations could be reproduced in patients who were admitted to an ICU. Thus, the impact of several weather conditions on short- and long-term mortality in critically ill patients has been examined for the first time.

## Materials and methods

### Study subjects

The study has been approved by the local ethics committee of the Jena University Hospital (Ethics Commission of the Friedrich-Schiller University Jena at the Medical Faculty). A large cohort of ICU patients was analyzed retrospectively. No formal sample size calculation for this observational retrospective study was performed. In all, 4321 patients who were admitted to the medical ICU of the Jena University Hospital between February 2004 and 31 December 2010 were recorded in this database. A subgroup of this cohort had been previously examined in other contexts [17]. For previous studies, medical history, clinical data, and standard laboratory parameters were documented [17]. Mortality data were collected by review of medical records in the in-hospital patient data management system (COPRA System GmbH, Berlin, Germany) and/or patient contact. The patient’s data were collected retrospectively but recorded prospectively in the patient data management system. All patient data was anonymized. According to the local ethics committee, no additional informed consent was necessary.

### Meteorological data

All meteorological information was obtained from the meteorological monitoring station of the Jena University of Applied Sciences (longitude 11°34′E/latitude 50°55′N/altitude 215 m). Starting on 27 February 2004 and ending 31 December 2010, the meteorological monitoring station collected mean air temperature, maximum air temperature, minimum air temperature, air pressure and humidity in Jena (Germany) once every hour. Based on this data, the exact meteorological data of the admission hour to the ICU was retrieved for each individual patient. Also, the change in climate conditions of the admission hour compared to 24 h before, for temperature, air pressure, and humidity was calculated. In addition, mean daily temperature, maximum, and minimum daily temperature were calculated for each admission. There are several different methods and definitions of “warm spells” and “cold spells” in the literature [18]. To identify the impact of extreme temperature deviations, the 1st and 99th percentile of the mean daily air temperature was calculated. There is no commonly accepted definition for sudden “cold” and “hot spells” [18]. For this study, two consecutive days with a minimum temperature under −7.9 °C defined a “cold spell” and two consecutive days with a maximum temperature above 28.9 °C defined a “hot spell”. Seasons were defined according to the meteorological classification: summer from June 1 to August 31; autumn from September 1 to November 30; winter from December 1 to February 28/29 and spring from March 1 to May 31 [19].

### Primary and secondary endpoints

The initial scores based on the Acute Physiology And Chronic Health Evaluation (APACHE-II) and Simplified Acute Physiology Score (SAPS-2) were calculated by the treating physician within 24 h after admission as previously reported. The primary endpoint of this study was the impact of meteorological parameters and sudden weather changes—defined as a sudden change in temperature, air pressure, and humidity—on all-cause ICU mortality. The secondary endpoint was long-term mortality. In a second step, all values were adjusted for APACHE-II or SAPS‑2 scores. In a subgroup analysis, the impact of all previously described weather conditions on the outcome of patients older than 75 years were studied. In addition, subgroup analyses for male and female, myocardial infarction and pneumonia were performed.

### Statistical analysis

Normally distributed data are expressed as mean ± standard deviation. Differences between independent groups were calculated using analysis of variance (ANOVA). Categorical data are expressed as numbers (percentage). Associations with intra-ICU mortality were analyzed with both univariable and multivariable logistic regression analysis to adjust for confounding factors for long-term mortality. For the multivariable regression model, a backward variable elimination was performed. The elimination criterion was a *p*-value of more than 0.10. Survival was depicted using a Kaplan–Meier curve. A *p*-value of < 0.05 was considered statistically significant. Analyses were performed with Microsoft® Excel 2010 for Windows (Microsoft, Redmond, WA, USA) and the IBM Statistical Package for the Social Sciences (SPSS) 23.0 for Windows (IBM, Armonk, NY, USA).

## Results

### Meteorological variables

**Fig. 1:**
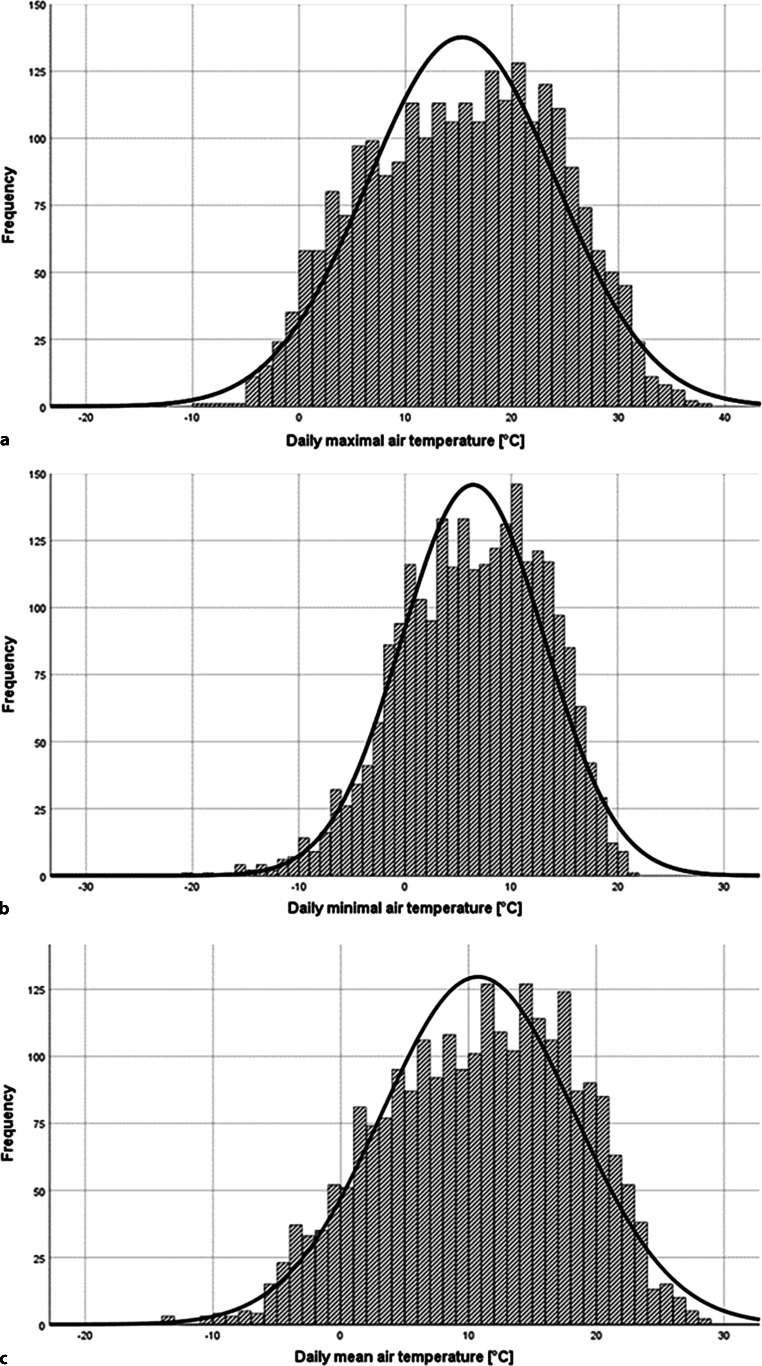
Statistical distribution of air temperature over the study period. Statistical distribution of air temperature over the study period 13 February 2004–31 December 2010. The frequencies of daily maximum (**a**), minimal (**b**) and mean temperatures are shown (**c**) [°C]. Additionally the normal distribution curve in *black*

**Fig. 2:**
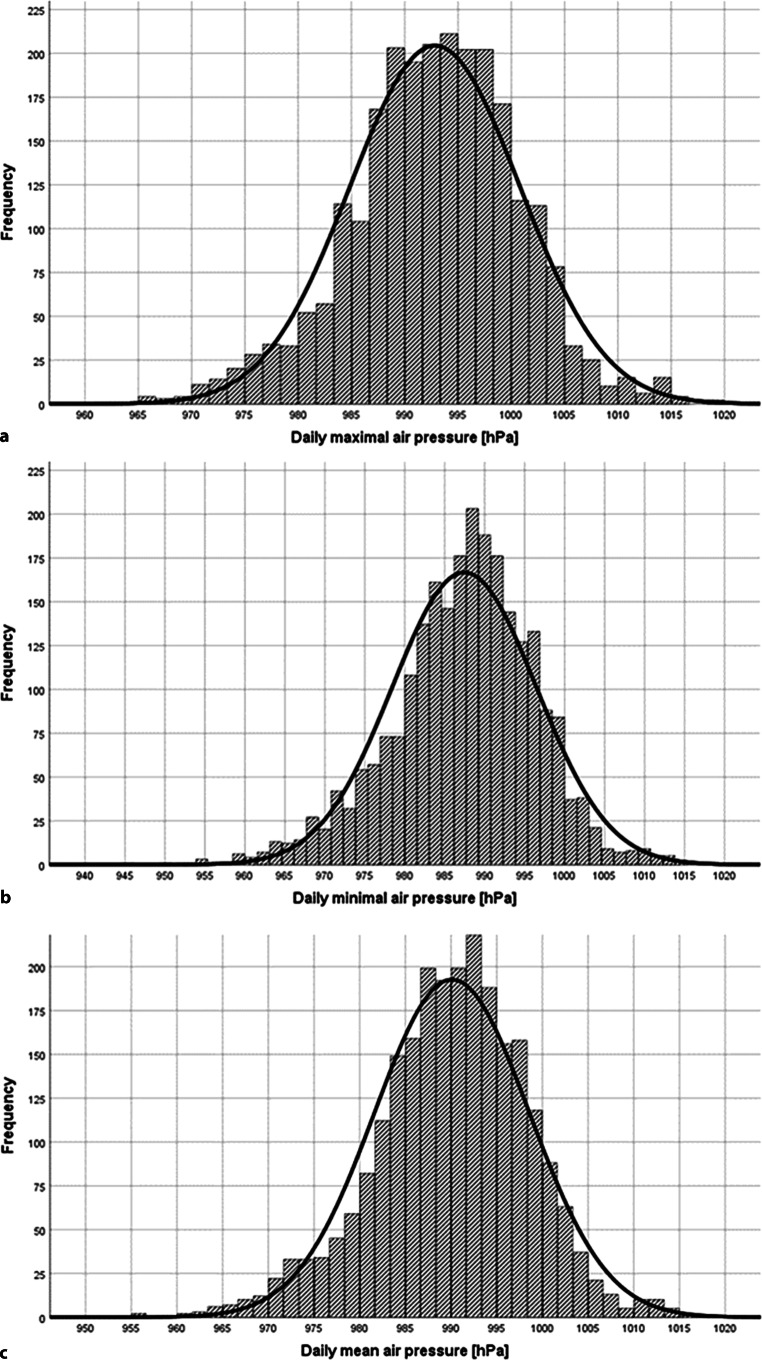
Statistical distribution of air pressure over the study period. Statistical distribution of air pressure over the study period 13 February 2004–31 December 2010. The frequencies of daily maximum (**a**), minimal (**b**) and mean pressures are shown (**c**) [hPa]. Additionally the normal distribution curve in *black*

**Fig. 3:**
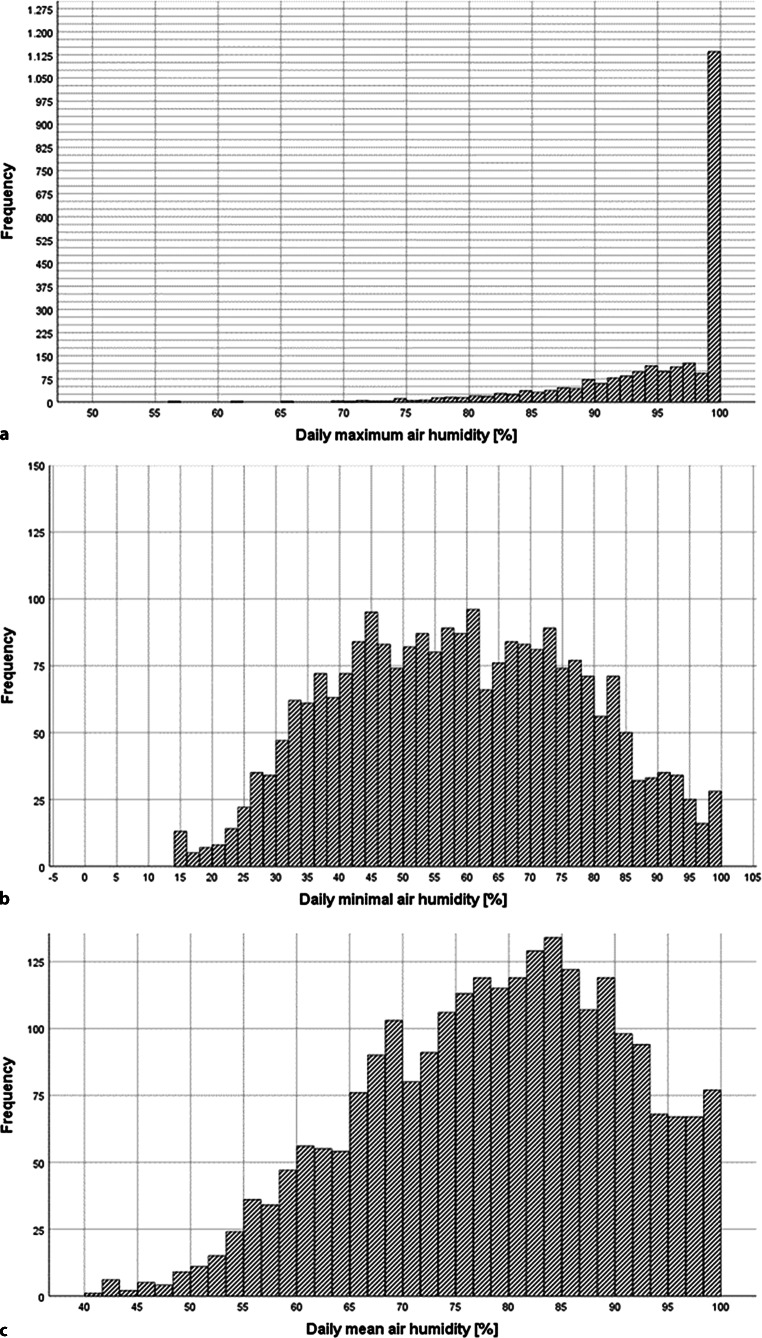
Statistical distribution of air humidity over the study period. Statistical distribution of air humidity over the study period 13 February 2004–31 December 2010. The frequencies of daily maximum (**a**), minimal (**b**) and mean (**c**) humidity are shown [%]. Additionally the normal distribution curve in *black*

Mean air temperature in Jena was 10.7 °C (± 7.5 °C SD), mean air pressure 990.1 hPa (± 8.5 hPa SD) and mean air humidity 78.6% (± 12.1% SD). The daily temperature ranged from −20.8 to 37.6 °C, while atmospheric pressure and relative humidity ranged from 945.2 to 1019 hPa and 15–100%, respectively (Tables 1, 2 and 3). Also, changes in 24 h in daily values of meteorological data were analyzed. Weather conditions are summarized in the supplemental materials (Figs. 1, 2 and 3).

**Table 1:** Mean values of mean, minimal and maximal air temperature, air pressure and air humidity

	Mean temperature [°C]	Minimal temperature [°C]	Maximal temperature [°C]
Mean	10.7	6.4	15.3
Median	11.3	6.8	15.7
SD	7.5	6.7	8.9
Minimum	−14.0	−20.8	−13.1
Maximum	28.6	21.0	37.6

Mean values of mean, minimal and maximal air temperature with median, standard deviation (SD), minimum and maximum values over the study period 13 February 2004–31 December 2010

**Table 2:** Mean values of mean, minimal and maximal air pressure

	Mean pressure [hPa]	Minimal pressure [hPa]	Maximal pressure [hPa]
Mean	990.1	987.4	992.8
Median	990.6	988.2	993.1
SD	8.5	9.0	8.0
Minimum	955.1	945.2	963.8
Maximum	1017.1	1014.5	1019.0

Mean values of mean, minimal and maximal air pressure with median, standard deviation (SD), minimum and maximum values over the study period 13 February 2004–31 December 2010

**Table 3:** Mean values of mean, minimal and maximal air humidity

	Mean humidity [%]	Minimal humidity [%]	Maximal humidity [%]
Mean	78.6	59.1	95.3
Median	79.7	59.0	98.2
SD	12.1	19.0	6.1
Minimum	40.0	15.0	56.8
Maximum	99.9	99.9	100.00

Mean values of mean, minimal and maximal air humidity with median, standard deviation (SD), minimum and maximum values over the study period 13 February 2004–31 December 2010

### Patients’ characteristics

In all, 4321 consecutive patients (age 66 ± 14 years, 61% male) were included. Nonsurvivors had significant higher values for age, lactate, SAPS‑2, APACHE-II, pCO_2_, ASAT (aspartate aminotransferase), ALAT (alanine aminotransferase), yGT (γ-glutamyltransferase), WBC (white blood count), urea, creatinine, and pCO_2_ (arterial carbon dioxide partial pressure) as well as reduced pO_2_ (arterial oxygen partial pressure, Table 4).

**Table 4:** Baseline characteristics and comparison of survivors and nonsurvivors

Parameter	Survivors (*n* = 3856) Mean ± SD	Nonsurvivors (*n* = 683) Mean ± SD	Overall cohort (*n* = 4539) Mean ± SD	*p*-value
Age [years]	66.7 ± 13.5	69.9 ± 12.4	66.6 ± 13.4	< 0.001
SAPS‑2	37.9 ± 17.0	61.9 ± 19.6	42.1 ± 19.7	< 0.001
APACHE-II	19.6 ± 8.8	29.9 ± 8.5	21.5 ± 9.6	< 0.001
Lactate [mmol/L]	2.0 ± 2.4	6.5 ± 5.9	2.6 ± 3.5	< 0.001
Hemoglobin [mmol/L]	7.8 ± 2.6	7.6 ± 5.8	7.8 ± 3.3	0.23
pO_2_ [kPa]	9.1 ± 1.9	8.6 ± 2.4	9.0 ± 2.0	< 0.001
pCO_2_ [kPa]	6.0 ± 1.6	7.5 ± 3.0	6.3 ± 2.0	< 0.001
ASAT [µmol/L]	3.5 ± 11.9	12.2 ± 32.2	5.0 ± 17.4	< 0.001
ALAT [µmol/L]	1.8 ± 6.3	5.4 ± 13.0	2.4 ± 8.0	< 0.001
γGT [µmol/L]	1.7 ± 2.4	2.2 ± 2.9	1.8 ± 2.5	< 0.001
WBC [×10^9^/L]	11.8 ± 10.1	15.7 ± 12.8	12.4 ± 10.6	< 0.001
Urea [mmol/L]	11.1 ± 9.9	17.6 ± 11.9	12.1 ± 10.5	< 0.001
Creatinine [µmol/L]	148.6 ± 150.4	211.0 ± 136.1	157.2 ± 150.0	< 0.001

*SAPS‑2* Simplified Acute Physiology Score, *APACHE-II* Acute Physiology, Age, Chronic Health Evaluation II, *ALAT* alanine aminotransferase, *ASAT* aspartate aminotransferase, *WBC* white blood count, *γGT* γ-glutamyltransferase, *pO*_*2*_ Arterial oxygen partial pressure, *pCO*_*2*_ Arterial carbon dioxide partial pressure; for all values, maximum results were recorded, except for pO_2_ with minimum values, *SD* standard deviation

### Main reasons for ICU admission

The ICU covered mainly nonsurgical intensive care medicine. As maximum care, reasons for ICU admissions were acute cardiologic emergencies (myocardial infarction, acute decompensated heart failure, and severe cardiac arrhythmia), respiratory failure, sepsis and septic shock, acute kidney injury, acute gastrointestinal bleeding, or acute liver failure. The ICU was competent for out-of-hospital cardiac arrests and for in-hospital cardiac arrests. The main reasons for admission for each season, cold and hot spells are summarized in Tables 5 and 6. There was no relevant difference between the seasons regarding the reasons for admission, but during hot spells, more cases of myocardial infarction were admitted.

**Table 5:** Main reason for all ICU admissions for each season

Admission	Spring (%)	Summer (%)	Autumn (%)	Winter (%)
Myocardial infarction	44.70	47.40	44.60	44.00
Acute cardiac decompensation	16.00	11.50	16.30	15.90
Sepsis	12.80	13.60	12.60	13.60
Pneumonia	13.80	12.60	9.80	15.50
OHCA	7.90	4.80	7	6.80
Pulmonary embolism	2.40	4.00	3.80	4.60

^a^*OHCA* out-of-hospital cardiac arrest, *ICU* intensive care unit

**Table 6:** Main reason for all ICU admissions for hot and cold spells

Admission	Hot spell (%)	Cold spell (%)
Myocardial infarction	49.50	36.80
Acute cardiac decompensation	13.40	17.10
Sepsis	11.10	9.20
Pneumonia	10.20	13.20
OHCA	4.60	6.60

^a^*OHCA* out-of-hospital cardiac arrest, *ICU* intensive care unit

### Impact of meteorological conditions on ICU mortality

In the next step, differences of survivors and nonsurvivors for absolute mean, minimum and maximum values of air temperature, air pressure, air humidity as well as dynamic changes of air temperature, air pressure and air humidity in 24 h were analyzed (see S1 Table). In brief, no significant differences between survivors and nonsurvivors for these meteorological parameters were found. In the present study, there was no significant effect of several meteorological parameters, neither regarding absolute values nor for sudden dynamic changes (Tables 7 and 8).

**Table 7:** Relationship between mortality on ICU and meteorological parameters (logistic regression)

Parameter	OR	CI (95%)	*p*-value
Mean air temperature	0.99	0.98–1.00	0.166
Minimum air temperature	0.99	0.98–1.01	0.217
Maximum air temperature	0.99	0.98–1.00	0.163
Mean air pressure	1.00	0.99–1.01	0.419
Minimum air pressure	1.00	0.99–1.01	0.371
Maximum air pressure	1.00	0.99–1.01	0.693
Mean air humidity	1.00	1.00–1.01	0.376
Minimum air humidity	1.00	1.00–1.01	0.407
Maximum air humidity	1.00	0.99–1.02	0.839
∆ 24 h Air temperature	0.98	0.96–1.01	0.214
∆ 24 h Air pressure	1.00	0.99–1.02	0.858
∆ 24 h Air humidity	1.00	0.99–1.00	0.104

*ICU* intensive care unit, *OR* odds ratio, *CI* confidence interval

**Table 8:** Relationship between mortality on ICU and meteorological parameters during summer adjusted for SAPS‑2 (logistic regression)

Parameter	HR	CI (95%)	*p*-value
Minimum air temperature	1.06	0.96–1.18	0.243
Mean air pressure	0.95	0.90–1.01	0.080
Minimum air pressure	0.96	0.91–1.01	0.098
Maximum air pressure	0.95	0.90–1.01	0.085

*ICU* intensive care unit, *HR *hazard ratio, *CI* confidence interval, *SAPS* Simplified Acute Physiology Score

### Cold and hot spells

**Fig. 4:**
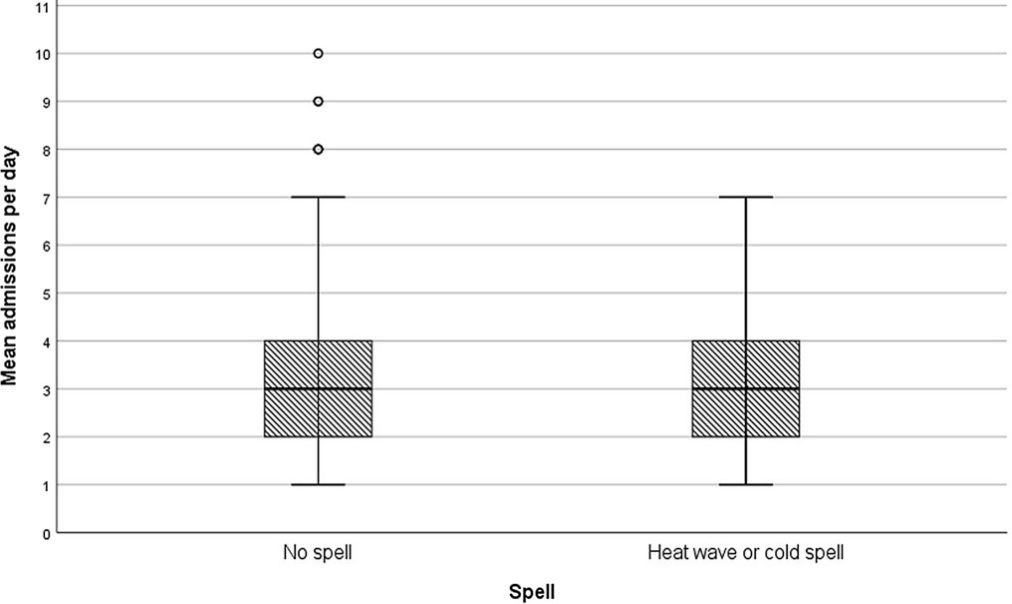
Difference in the mean number of admissions per day. *No spell* day does not meet criteria for warm or cold spell (see “Methods” section); *Warm or cold spell* day does meet criteria for either heat wave or cold spell. *n* = 2430, *p* = 0.225

In total, 58 days with a “cold” and 143 days with a “warm spell” could be identified. On these days 222 “warm spell” and 80 “cold spell” patients, respectively, were admitted to ICU. Both had no significant difference in ICU mortality compared to days without an extreme temperature deviation (*p* = 0.154 for warm spells and *p* = 0.744 for cold spells). The mean and median admission rates showed no statistically significant difference between days with cold or hot spells on the one, and days without on the other hand (Fig. 4).

### Impact of seasonal variations on ICU and long-term mortality

**Fig. 5:**
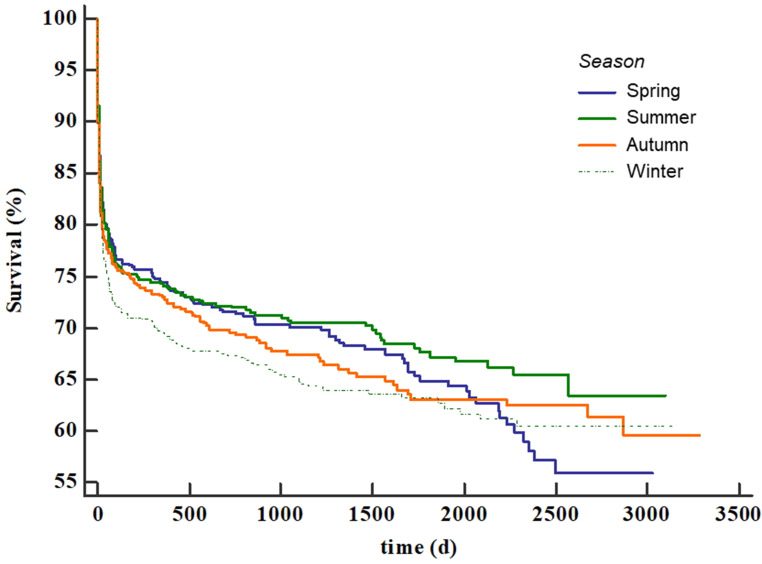
Kaplan–Meier for long-term survival of different seasons. *d* days

During summer, air pressure and minimum air temperature demonstrated a significant impact on ICU mortality. For mean (OR 0.96, CI 0.93–0.99, *p* = 0.016), minimum (OR 0.96, CI 0.93–0.99, *p* = 0.021) and maximum (OR 0.96, CI 0.92–0.99, *p* = 0.018) air pressure and minimum air temperature (OR 1.08, CI 1.1–1.12, *p* = 0.017), a significant effect could be observed. All other investigated meteorological conditions had no effect (S2 Table). However, after correction for SAPS‑2, statistical significance for air pressure vanished (see S3 Table). For winter, no impact on mortality could be assessed (see S4 Table). The Kaplan–Meier curve for different seasons showed no significant difference in long-term mortality (log-rank test *p* = 0.18, Fig. 5).

### Impact of meteorological conditions on ICU mortality of patients with myocardial infarction or pneumonia

A total of 1954 patients with myocardial infarction were analyzed (see S5 Table). None of the investigated weather conditions influenced ICU mortality (1806 survivors, 149 nonsurvivors). For pneumonia, 548 patients were identified (S6 Table). A sudden change in air humidity was significantly associated with increased mortality (OR 0.99, CI 0.97–1.00, *p* = 0.026), but after correction for SAPS‑2, no statistical effect remained (OR 0.98, CI 0.97–1.00, *p* = 0.055).

### Impact of meteorological conditions on ICU mortality of old intensive care patients

For subgroup analysis, all values for patients older than 75 years were calculated. We analyzed 1221 patients (28%). In this subgroup, there were 995 survivors and 226 nonsurvivors. In all, no significant impact was found for either absolute weather conditions or of dynamic changes in 24 h on ICU mortality of old intensive care patients (see S7 Table).

### Impact of meteorological conditions on ICU mortality regarding male or female patients

Finally, the cohort was divided into a female and a male subgroup. For both groups, the impact of the predefined weather parameters was analyzed. As demonstrated for female (S8 Table) and for male patients (S9 Table), there was no significant impact of several weather conditions in the two groups.

## Discussion

This investigation found no impact of meteorological conditions on the outcome of critically ill patients. Thus, this study contrasts with many observational and epidemiologic studies in different countries that found a significant relationship between the weather and several acute diseases or outcome parameters.

Large epidemiologic studies in the US were able to demonstrate a relationship between mortality and sudden onset of rising temperatures and decreased air pressure. Several diseases had been investigated in detail. In many countries, an acute myocardial infarction occurs more often during winter and spring and significantly less during summer (Japan [9], US [10], Sicily [11], Greece [12], and Iran [20]). On the contrary, in Germany, the incidence of acute myocardial infarction rises during heat waves [1]. This is in line with results from Canada, where myocardial infarction occurs in young women significantly more often during warm seasons [21]. In Brazil, both extraordinarily high and low temperatures were associated with a rise in cardiovascular mortality [22]. Highly urbanized cities seem to be more susceptible for heat waves [23]. Colder temperatures are more often accompanied by increased air pollution with a higher NO_2_ concentration, which could explain the increased cardiovascular mortality in China, for example [24]. In this investigation, there was a rise for myocardial infarction during heat waves, but no effect on short- or long-term mortality could be found.

Weather influences the admission rates for respiratory diseases, for example, in Spain during cold, dry weather or during humid, hotter weather [25]. Japanese studies found an increased incidence combined with a poorer prognosis for ischemic stroke during winter or spring [26]. In the cohort of this study, there were no seasonal differences in the reasons for admission to intensive care. Furthermore, the composition of the reasons for admission during cold spells and heat waves was also very similar, but in line with the existing data, there were more cases of myocardial infarction during hot spells. However, no increased mortality could be observed. In northern Europe, an impact of weather conditions on mortality could be identified too. Both cold temperatures during winter and heatwaves during summer increased mortality, with a relatively stronger effect for heat [4]. Southern Europe showed a similar pattern with both very low and very high ambient temperatures raising mortality [27]. However, the present study found no impact of extreme temperatures on ICU or long-term mortality.

It could be hypothesized that weather observations could be valid only for these special regions with their specific meteorological conditions. In other words, Central Europa might be inappropriate to show any weather-related effects at all. However, this hypothesis can be refuted: For the city of Jena, Rakers et al. had already investigated the effect of rapid weather changes. They found a significant impact on the incidence of ischemic stroke [28]. The same investigators demonstrated that low atmospheric pressure and high relative air humidity increased the risk for epileptic seizures, whereas high ambient temperatures decreased the risk for seizure [29]. Recently, Ostendorf et al. showed a significant impact of rapid weather changes on the incidence of cardiovascular diseases in the emergency department for Leipzig [7]. As already referred above, there is sufficient data for Augsburg (Germany) showing significant effects of heat on the incidence of myocardial infarction. Furthermore, in central Europe, Plavcová et al. analyzed retrospective data of a 1.2 million population over a 16-year period (1994–2009) and found a significant effect of sudden air pressure changes on cardiovascular mortality, a significant excess in cardiovascular disease mortality and a significant rise in hospital admissions [13]. Also, cold stress was identified as a major risk factor for mortality in Central Europe [15]. In the same region, both cold and hot temperatures were associated with an increase in the mortality from ischemic heart disease with an age-dependent peak.

In Vienna, outer air temperature affects the body temperature of patients suffering from cardiac arrest. However, these alternations did not result in a significant difference in neurological outcome [30]. Similarly, regarding only patients with out-of-hospital cardiac arrest (OHCA), Nedelea et al. found a seasonal pattern for the incidence, but no increased mortality in Romania.

While patients over 65 years seemed to be more affected by hot waves, younger patients were more sensitive to cold spells [15]. The present study found no significant increased susceptibility of older patients to weather conditions. This result is consistent with previous studies that also found no influence of patient age on the outcome of ventilated intensive care patients [31].

Another important issue is the definition of “hot” and “cold spells”. There is no commonly accepted definition of heat and cold waves [18]. There are many different climate zones with different social adaptation methods. Compared to other geographical regions, the climate in Jena can be defined as “moderate” [32]. Therefore, the present study chooses a very strict definition, using only temperatures above or under the 1st or 99th percentile. These extreme weather deviations do not lead to an increased admission rate to ICU or to an increased ICU mortality.

In summary, there is sufficient data showing a significant impact of meteorological conditions on the incidence of several diseases and mortality in Jena and in Central Europe. Therefore, meteorological data from Jena can be considered very suitable for the present investigation. As stated before, no relationship between age, sex, cold or heat, air pressure or air humidity, on the one hand, and mortality, on the other hand, could be found in this investigation.

Another difference between this investigation and the cited study results might be that many studies used epidemiologic data. Consequently, many deaths may occur “unnoticed” outside the hospital without reaching any intensive care unit. This may explain a lack of significant meteorological impact on ICU mortality. However, many retrospective studies demonstrated a rise in hospital admissions during extreme weather conditions [11–13].

This study suffers from limitations. It is retrospective and reflects only a single-center experience covering nonsurgical intensive care, excluding cases of acute trauma. Another issue is that the timing we used to set the temperature was at the time of admission to the ICU. Other models, such as the onset of the symptoms leading to the admission, might be more appropriate, but are difficult to capture. Our analysis compensates for this with the effects of the seasons and the “spells”, which we examined and which each represent several days—and could not demonstrate any effect.

As discussed above, a non-insignificant part of meteorological conditions depends on geography. The impact of weather conditions was evaluated for Jena (Germany), which might be considered representative of central-western Europe. Results may, of course, be different for regions with another type of climate. Most patients reached the hospital by ground-based rescue equipment and in moderate transport time. This, too, cannot necessarily be transferred to other care structures. A global multicenter approach is warranted for statements that are more general.

## Conclusions

In central-western Europe, no significant impact of sudden weather changes or extreme weather conditions on outcome parameters of critically ill patients could be found.

## Supplementary Information

Relationship between mortality on ICU and different meteorological parameters
