# Supplementary material for: No impact of weather conditions on the outcome of intensive care unit patients
Source: Wien Med Wochenschr. 2021 Mar 18;172(1-2):40–51. doi: 10.1007/s10354-021-00830-0 (PMC8837525; doi:10.1007/s10354-021-00830-0)
Supplement: Supplementary file 1 — Relationship between mortality on ICU and different meteorological parameters [file 10354_2021_830_MOESM1_ESM.docx]

**S1 Table: Weather characteristics and comparison of survivors and non-survivors.** *Mean values with Standard deviation.*

| Parameter | Survivors *Mean±SD* | Non-Survivors *Mean±SD* | Overall cohort *Mean±SD* | *p*-value |
| --- | --- | --- | --- | --- |
| Δ Temperature 24h [C°] | 0.1±3.3 | -0.1±3.3 | 0.1±3.3 | 0.214 |
| Δ Pressure 24h [hPA] | 0±6 | 0±6.2 | 0±6a | 0.858 |
| Δ Humidity 24h [%] | 0.1±16.3 | -1.1±15.1 | -0.1±16.1 | 0.104 |
| Mean Temperature [C°] | 10.9±7.5 | 10.4±7.6 | 10.8±7.5 | 0.166 |
| Minimum Temperature [C°] | 6.5±6.6 | 6.2±6.8 | 6.5±6.6 | 0.217 |
| Maximum Temperature [C°] | 15.5±8.8 | 14.9±8.9 | 15.4±8.9 | 0.163 |
| Mean Pressure [hPA] | 990.4±8.5 | 990.1±9.1 | 990.3±8.6 | 0.419 |
| Minimum Pressure [hPA] | 987.8±9.1 | 987.4±9.6 | 987.7±9.2 | 0.371 |
| Maximum Pressure [hPA] | 993.1±8 | 993±8.6 | 993.1±8.1 | 0.693 |
| Mean Humidity [%] | 78.3±12.5 | 78.8±11.9 | 78.4±12.5 | 0.376 |
| Minimum Humidity [%] | 58.8±19.3 | 59.5±18.6 | 58.9±19.2 | 0.407 |
| Maximum Humidity [%] | 95.3±6.3 | 95.3±5.6 | 95.3±6.2 | 0.839 |

**S2 Table: Relationship between mortality on ICU and meteorological parameters during summer (logistic regression).**

| Parameter | OR | CI (95%) | *p*-value |
| --- | --- | --- | --- |
| Mean air temperature | 1.02 | 0.97-1.08 | 0.387 |
| Minimum air temperature | 1.08 | 1.1-1.12 | 0.017 |
| Maximum air temperature | 1.01 | 0.97-1.05 | 0.803 |
| Mean air pressure | 0.96 | 0.93-0.99 | 0.016 |
| Minimum air pressure | 0.96 | 0.93-0.99 | 0.021 |
| Maximum air pressure | 0.96 | 0.92-0.99 | 0.018 |
| Mean air humidity | 1.00 | 0.99-1.02 | 0.840 |
| Minimum air humidity | 1.00 | 0.99-1.02 | 0.585 |
| Maximum air humidity | 1.00 | 0.97-1.02 | 0.727 |
| Δ24h Air temperature | 0.97 | 0.92-1.02 | 0.212 |
| Δ24h Air pressure | 1.00 | 0.96-1.05 | 0.853 |
| Δ24h Air humidity | 1.00 | 0.99-1.01 | 0.727 |

**S3 Table Relationship between mortality on ICU and meteorological parameters during summer adjusted for SAPS-2 (logistic regression)**

| Parameter | HR | CI (95%) | *p*-value |
| --- | --- | --- | --- |
| Minimum air temperature | 1.06 | 0.96-1.18 | 0.243 |
| Mean air pressure | 0.95 | 0.90-1.01 | 0.080 |
| Minimum air pressure | 0.96 | 0.91-1.01 | 0.098 |
| Maximum air pressure | 0.95 | 0.90-1.01 | 0.085 |

**S4 Table. Relationship between mortality on ICU and meteorological parameters during winter (logistic regression).**

| Parameter | OR | CI (95%) | *p*-value |
| --- | --- | --- | --- |
| Mean air temperature | 1.00 | 0.97-1.04 | 0.824 |
| Minimum air temperature | 1.00 | 0.96-1.03 | 0.884 |
| Maximum air temperature | 1.00 | 0.67-1.04 | 0.821 |
| Mean air pressure | 1.00 | 0.99-1.02 | 0.685 |
| Minimum air pressure | 1.00 | 0.99-1.02 | 0.823 |
| Maximum air pressure | 1.01 | 0.99-1.02 | 0.490 |
| Mean air humidity | 1.00 | 0.98-1.02 | 0.806 |
| Minimum air humidity | 1.00 | 0.98-1.01 | 0.754 |
| Maximum air humidity | 0.99 | 0.96-1.02 | 0.490 |
| Δ24h Air temperature | 0.97 | 0.92-1.02 | 0.254 |
| Δ24h Air pressure | 1.00 | 0.98-1.02 | 0.99 |
| Δ24h Air humidity | 0.99 | 0.98-1.01 | 0.279 |

**S5 Table. Relationship between mortality on ICU and meteorological parameters in a subgroup analysis for patients with myocardial infarction (logistic regression).**

| Parameter | OR | CI (95%) | *p*-value |
| --- | --- | --- | --- |
| Mean air temperature | 1.01 | 0.99-1.03 | 0.458 |
| Minimum air temperature | 1.01 | 0.98-1.03 | 0.646 |
| Maximum air temperature | 1.01 | 0.99-1.03 | 0.406 |
| Mean air pressure | 0.98 | 0.97-1.00 | 0.110 |
| Minimum air pressure | 0.99 | 0.97-1.00 | 0.122 |
| Maximum air pressure | 0.99 | 0.97-1.01 | 0.148 |
| Mean air humidity | 1.00 | 0.99-1.03 | 0.928 |
| Minimum air humidity | 1.00 | 0.99-1.01 | 0.450 |
| Maximum air humidity | 1.02 | 0.99-1.05 | 0.220 |
| Δ24h Air temperature | 0.98 | 0.93-1.04 | 0.520 |
| Δ24h Air pressure | 0.98 | 0.95-1.01 | 0.156 |
| Δ24h Air humidity | 1.00 | 0.99-1.01 | 0.718 |

**S6 Table. Relationship between mortality on ICU and meteorological parameters in a subgroup analysis for patients with pneumonia (logistic regression).**

| Parameter | OR | CI (95%) | *p*-value |
| --- | --- | --- | --- |
| Mean air temperature | 1.01 | 0.98-1.04 | 0.483 |
| Minimum air temperature | 1.01 | 0.98-1.04 | 0.661 |
| Maximum air temperature | 1.01 | 0.99-1.03 | 0.403 |
| Mean air pressure | 1.01 | 0.98-1.03 | 0.624 |
| Minimum air pressure | 1.01 | 0.99-1.03 | 0.491 |
| Maximum air pressure | 1.01 | 0.98-1.03 | 0.610 |
| Mean air humidity | 0.99 | 0.98-1.01 | 0.216 |
| Minimum air humidity | 0.99 | 0.98-1.00 | 0.172 |
| Maximum air humidity | 1.1 | 0.98-1.04 | 0.762 |
| Δ24h Air temperature | 1.05 | 0.98-1.12 | 0.145 |
| Δ24h Air pressure | 1.00 | 0.76-1.04 | 0.779 |
| Δ24h Air humidity | 0.99 | 0.97-1.00 | 0.026 |

**S7 Table. Subgroup analysis of the relationship between mortality on ICU and meteorological parameters in old intensive care patients (logistic regression).**

| Parameter | OR | CI (95%) | *p*-value |
| --- | --- | --- | --- |
| Mean air temperature | 1.00 | 0.98-1.02 | 0.888 |
| Minimum air temperature | 1.00 | 0.98-1.02 | 0.820 |
| Maximum air temperature | 1.00 | 0.98-1.02 | 0.952 |
| Mean air pressure | 0.99 | 0.97-1.00 | 0.987 |
| Minimum air pressure | 0.99 | 0.98-1.01 | 0.257 |
| Maximum air pressure | 0.99 | 0.97-1.01 | 0.156 |
| Mean air humidity | 1.00 | 0.99-1.02 | 0.587 |
| Minimum air humidity | 1.00 | 0.99-1.01 | 0.806 |
| Maximum air humidity | 1.01 | 0.99-1.04 | 0.304 |
| Δ24h Air temperature | 1.02 | 0.97-1.06 | 0.470 |
| Δ24h Air pressure | 0.98 | 0.96-1.00 | 0.981 |
| Δ24h Air humidity | 1.00 | 0.99-1.01 | 0.487 |

**S8 Table. Subgroup analysis of the relationship between mortality on ICU and meteorological parameters in female patients (logistic regression).**

| Parameter | OR | CI (95%) | *p*-value |
| --- | --- | --- | --- |
| Mean air temperature | 0.99 | 0.97-1.01 | 0.292 |
| Minimum air temperature | 0.99 | 0.97-1.01 | 0.422 |
| Maximum air temperature | 0.99 | 0.98-1.01 | 0.265 |
| Mean air pressure | 1.00 | 0.98-1.91 | 0.585 |
| Minimum air pressure | 1.00 | 0.98-1.01 | 0.997 |
| Maximum air pressure | 1.00 | 0.98-1.01 | 0.633 |
| Mean air humidity | 1.00 | 0.99-1.01 | 0.760 |
| Minimum air humidity | 1.00 | 0.99-1.01 | 0.790 |
| Maximum air humidity | 1.00 | 0.97-1.02 | 0.631 |
| Δ24h Air temperature | 0.98 | 0.95-1.03 | 0.457 |
| Δ24h Air pressure | 1.01 | 0.98-1.03 | 0.643 |
| Δ24h Air humidity | 0.99 | 0.98-1.00 | 0.115 |

**S9 Table. Subgroup analysis of the relationship between mortality on ICU and meteorological parameters in male patients (logistic regression).**

| Parameter | OR | CI (95%) | *p*-value |
| --- | --- | --- | --- |
| Mean air temperature | 0.99 | 0.98-1.01 | 0.358 |
| Minimum air temperature | 0.99 | 0.98-1.01 | 0.353 |
| Maximum air temperature | 0.99 | 0.98-1.01 | 0.376 |
| Mean air pressure | 1.00 | 0.98-1.01 | 0.552 |
| Minimum air pressure | 1.00 | 0.98-1.01 | 0.393 |
| Maximum air pressure | 1.00 | 0.99-1.01 | 0.905 |
| Mean air humidity | 1.00 | 1.00-1.01 | 0.371 |
| Minimum air humidity | 1.00 | 1.00-1.01 | 0.393 |
| Maximum air humidity | 1.01 | 0.99-1.03 | 0.508 |
| Δ24h Air temperature | 0.98 | 0.95-1.02 | 0.325 |
| Δ24h Air pressure | 1.00 | 0.98-1.02 | 0.878 |
| Δ24h Air humidity | 1.00 | 0.99-1.00 | 0.399 |
